# Reviewers List

**DOI:** 10.1002/rmb2.12311

**Published:** 2020-01-12

The publication of invaluable papers in the Reproductive Medicine and Biology depends on the prompt, careful review of submitted manuscripts. We would like to thank the Editorial Board members, the Editorial Advisory Board members and the following experts for reviewing manuscripts from November 1, 2018, to October 31, 2019.

Toshiyasu Amano

Yasuhisa Araki

Yoshimasa Asada

Yoshiyuki Asai

Atsushi Azumaguchi

Tsuyoshi Baba

Koji Chiba

Masashi Deguchi

Toshihiro Fujiwara

Aisaku Fukuda

Toshio Hamatani

Tetsuaki Hara

Shu Hashimoto

Osamu Hiraike

Yuji Hirao

Kenichiro Hiraoka

Kentaro Ichioka

Hideki Igarashi

Takeshi Iwasa

Akira Iwase

Kenta Izaki

Haruhiko Kanasaki

Naoko Kimura

Michio Kitajima

Tomoya Kitajima

Yoshitomo Kobori

Jin Kumagai

Keiji Kuroda

Ryo Maekawa

Hirotaka Masuda

Naojiro Minami

Yasuyuki Mio

Satoshi Mizuno

Tetsunori Mukaida

Motoo Nabeta

Yoshiaki Nakamura

Mikiya Nakatsuka

Kaei Nasu

Makoto Orisaka

Manabu Satoh

Makio Shozu

Tetsuji Soda

Tatsuya Suzuki

Kazumasa Takahashi

Yasushi Takai

Isao Tamura

Atsushi Tanaka

Kentaro Tanemura

Pei‐Shiue Tsai

Isao Tsuji

Tateki Tsutsui

Sayaka Uchida

Satoshi Ueno

Takashi Umehara

Tomohiko Wakayama

Hideaki Watanabe

Yuri Yamamoto

Yasuhisa Yamashita

Toshiyuki Yasui

Masaki Yokoo

Hiroaki Yoshida

Shosei Yoshida

Osamu Yoshino

Yasushi Yumura

